# The In Vitro and In Vivo Effect of Lipoxygenase Pathway Inhibitors Nordihydroguaiaretic Acid and Its Derivative Tetra-*O*-methyl Nordihydroguaiaretic Acid against Brucella abortus 544

**DOI:** 10.4014/jmb.2207.07026

**Published:** 2022-08-29

**Authors:** Alisha Wehdnesday Bernardo Reyes, Heejin Kim, Tran Xuan Ngoc Huy, Trang Thi Nguyen, Wongi Min, Dongho Lee, Jin Hur, John Hwa Lee, Suk Kim

**Affiliations:** 1 Department of Veterinary Paraclinical Sciences, College of Veterinary Medicine, University of the Philippines Los Baños, College, Laguna 4031, Philippines; 2 Institute of Animal Medicine, College of Veterinary Medicine, Gyeongsang National University, Jinju 52828, Republic of Korea; 3 College of Medicine, Inje University, Busan, 47392, Republic of Korea; 4 College of Veterinary Medicine, Chonbuk National University, Iksan 54596, Republic of Korea

**Keywords:** 5-Lipoxygenase, *Brucella abortus*, leukotriene B4, thromboxane A2, splenic proliferation

## Abstract

This study investigated the contribution of lipoxygenase (LOX) inhibitors, nordihydroguaiaretic acid (NDGA), tetra-*O*-methyl nordihydroguaiaretic acid (M_4_N) and zileuton (ZIL), and thromboxane A2 (TXA_2_) inhibitor 4,5-diphenylimidazole (DPI) in the proliferation of *Brucella abortus* infection. None of the compounds affected the uptake of *Brucella* into the macrophages. We determined the effect of neutralizing leukotriene B4 (LTB4) receptor and showed that the uptake of the bacteria was inhibited at 30 min post-infection. M_4_N treatment attenuated intracellular survival of *Brucella* at 2 h post-incubation but it was not observed in the succeeding time points. DPI treatment showed reduced survival of *Brucella* at 24 h post-incubation while blocking LTB4 receptor was observed to have a lower intracellular growth at 48 h post-incubation suggesting different action of the inhibitors in the course of the survival of *Brucella* within the cells. Reduced proliferation of the bacteria in the spleens of mice was observed in animals treated with ZIL or DPI. Increased serum cytokine level of TNF-α and MCP-1 was observed in mice treated with M_4_N or ZIL while a lower IFN-γ level in ZIL-treated mice and a higher IL-12 serum level in DPI-treated mice were observed at 7 d post-infection. At 14 d post-infection, ZIL-treated mice displayed reduced serum level of IL-12 and IL-10. Overall, inhibition of 5-LOX or TXA_2_ or a combination therapy promises a potential alternative therapy against *B. abortus* infection. Furthermore, strong ligands for LTB4 receptor could also be a good candidate for the control of *Brucella* infection.

## Introduction

Brucellosis is an important zoonotic disease that affects domestic livestock and wildlife, the second most transmissible zoonotic disease worldwide next to rabies, a serious health hazard but its public health concern has been neglected in many countries, a notifiable disease, and considered a bio-threatening (category B) agent [1]. This zoonotic infection is caused by *Brucella*, four species are known to infect humans among which majority of the cases are caused by *B. abortus* and *B. melitensis* [2]. *Brucella* has been reported to possess five virulence factors necessary for infection and survival within the host including cyclic β-glucan, virB T4SS, pathogen-associated molecular patterns (PAMPs), two component sensory and regulatory system BvrS/BvrR and lipopolysaccharide (LPBS); as well as it requires four steps to successfully establish an infection to its host such as adherence, invasion, establishment and dissemination [3]. Although *Brucella* primarily infects and replicates inside phagocytic cells such as macrophages and dendritic cells, it has also the ability to replicate in a wide range of mammalian cell types including endothelial cells, fibroblasts, epithelial cells and microglia [4]. *B. abortus* has been reported to invade murine fibroblasts NIH3T3, green monkey kidney Vero cells, human epitheloid cell line HeLa, Madin-Darby bovine kidney cells and baby hamster kidney cells [5].

Arachidonic acid (AA), being an integral constituent of biological cell membrane, is a polyunsaturated fatty acid (PUFA) essential for normal health as it is involved in the proper functioning of all cells especially in nervous system, skeletal muscle and immune system [6]. AA is obtained from food or via desaturation and chain elongation of linoleic acid (LA) and it is a precursor in the biosynthesis of biologically active FA mediators metabolized by cyclooxygenases (COXs), lipoxygenases (LOXs) and cytochrome P450 (CYP) enzymes via phospholipase A2 (PLA2) [6, 7]. We previously reported that AA was highly toxic to *B. abortus* 544 [8]. AA is converted to unstable endoperoxides by COX from which prostaglandins (PG), prostacyclin (PC) and thromboxanes (TX) are derived [9]. On the other hand, 5-lipoxygenase (5-LOX) initiates formation of leukotrienes from AA to produce leukotriene A4 (LTA4) from which its subsequent hydrolysis results in the formation of LTB4 [9].

Nordihydroguaiaretic acid (NDGA), an antioxidant, phenolic compound and main metabolite found in extracts from desert shrub *Larrea tridentata*, is a natural LOX inhibitor and has been reported to inhibit the activity of 5-LOX in vitro [10]. 5-LOX has been targeted to discover compounds for the purpose of inhibiting pathophysiology associated with leukotrienes formation [11]. NDGA is being evaluated for the treatment of a variety of illnesses that include diabetes, pain, inflammation, infertility and gallbladder and kidney stones [10]. Furthermore, this phenolic compound has been reported to exhibit antimicrobial activity possibly via targeting bacterial cell membrane [12]. Its antiviral effect, on the other hand, as well as its methylated derivative tetra-*O*-methyl nordihydroguaiaretic acid (M_4_N), has been associated with its ability to disturb lipid metabolism via interfering with the sterol regulatory element-binding proteins (SREBP) pathway [10]. SREBP are lipid synthetic transcription factors for cholesterol and fatty acid synthesis, and reported to play essential roles in coupling lipid metabolism with several biological, physiological and pathological processes or metabolic diseases [13, 14]. A synthetic chiral compound of NDGA, nordy, was found to diminish the expression of chemokine receptor 4 (CXCR4) and formyl peptide receptor (FPR) in glioma cells, both are G-protein-coupled receptor (GPCR), which might indirectly suggest the anticancer activities of NDGA [15, 16]. We also recently reported the beneficial effects of CXCR4 and FPR2 inhibition in the progression of *B. abortus* 544 infection [17, 18]. Here we explore the effect of NDGA and M_4_N on *B. abortus* 544 infection in a murine professional phagocyte cell line using RAW264.7 cells as well as in a murine model using ICR mice.

## Materials and Methods

### Materials and Chemicals

The following reagents were supplied by Sigma-Aldrich (USA): NDGA (molecular weight, MW: 302.36 g/mol), M_4_N (MW: 358.47 g/mol), zileuton (ZIL) (MW: 236.29 g/mol) 4,5-diphenylimidazole (DPI) (MW: 220.27 g/mol), 1% penicillin-streptomycin (10,000 U penicillin and 10 mg streptomycin/ml), streptomycin solution (1 mg/ml), 3-(4,5-dimethylthiazol-2-yl)-2,5-diphenyltetrazolium bromide (MTT), ethanol and dimethyl sulfoxide (DMSO). LTB4 receptor (BLT2) polyclonal antibody was purchased from Enzo Life Sciences, Inc. (USA), RPMI 1640 was from Life Technologies Corporation (USA), fetal bovine serum (FBS) from Thermo Fisher Scientific (USA), GPT (Mouse) ELISA kit was from BioVision Incorporated (USA) and BD cytometric bead array (CBA) mouse inflammation kit was purchased from BD Biosciences (USA). Agar was purchased from Yakuri Pure Chemicals Co., Ltd (Japan), *Brucella* broth from Becton Dickinson (USA). The virulent smooth strain *B. abortus* 544 biovar 1 (ATCC 23448) was kindly provided by the Laboratory of Bacteriology Division in Animal and Plant Quarantine Agency in Korea and cultivated in broth for 2 d with shaking (180 ×*g*) prior to infection assays or in 2% agar for at least 3 d at 37°C to determine the number of colony forming units (CFUs). The murine phagocytic cell line, RAW264.7 cells (TIB-71, USA), was cultured in RPMI 1640 supplemented with 10% heat-inactivated FBS and 1%penicillin-streptomycin in 5% CO_2_ at 37°C. Prior to all in vitro procedures, antibiotics were removed and appropriate controls was used in reference to the percentage (w/v) of the diluent in each compound.

### Cell Viability

RAW264.7 cells were seeded in a 96-well culture plate at a concentration of 1 × 10^5^ cells per well incubated overnight. Cell medium was changed to fresh medium without antibiotics containing different concentrations of NDGA (0, 0.1, 0.5, 1, 10, 20, 30, and 50 μM), M_4_N (0, 0.1, 0.5, 1, 10, 20, 30, and 50 μM), ZIL (0, 0.1, 0.5, 1, 10, 20, 30, 50 μM) or DPI (0, 0.1, 0.5, 1, 10, 20, 30, 50 μM) incubated for 48 h. The vehicle control for NDGA, M_4_N and ZIL was 0.1% DMSO while for DPI was 0.1% ethanol. Cell viability was computed using MTT assay. Briefly, cells were washed, the medium was changed to RPMI 1640 containing 5 mg/ml MTT solution and incubated for at least 2 h. The medium was then removed and 150 μl DMSO was added. After 15 mins, the absorbance was measured at an optical density (OD) of 540 nm. The highest concentration for each compound that did not affect the cell viability was used for the succeeding experiments: NDGA (1 μM), M_4_N (1 μM), ZIL (50 μM), or DPI (50 μM).

### Bacterial Internalization and Intracellular Growth Assays

Overnight culture of RAW264.7 cells seeded in a 96-well culture plate at a concentration of 1 × 10^5^ cells per well were used to determine the bacterial adhesion, internalization and intracellular growth efficiencies. For internalization assay, cells were incubated with NDGA, M_4_N, ZIL or DPI for at least 4 h. Cells were washed and then infected with *B. abortus* at a multiplicity of infection (MOI) of 50 in RPMI 1640 containing 10% FBS. The cells were then centrifuged at 200 ×*g* for 5 min and incubated for 0, 15 and 30 min. At indicated time points, cells were washed and then incubated with RPMI 1640 containing 10% FBS and 100 μg/ml gentamicin for 30 min. The cells were then washed and lysed using distilled water. Serial dilutions were plated onto *Brucella* agar to determine the CFUs per ml. For intracellular replication assay, cells were infected with *Brucella* for 1 h and then incubated with fresh medium containing treatment and 100 μg/ml gentamicin for 1 h. The cells were washed and further incubated with the treatment but reduced concentration of gentamicin (30 μg/ml) for a total of 2, 24, and 48 h incubation.

### Mouse Studies

All animal experiments were performed according to procedures approved by the Animal Ethical Committee of Chonbuk National University (Authorization Number CBNU-2018-00374). Seven-week-old female ICR mice were randomly distributed into six groups of at least six mice each group. The mice were acclimatized first for one week prior to treatment in metabolic cages. Oral treatment of NDGA (1 μM), M_4_N (1 μM), ZIL (50 μM) or DPI (50 μM) was done using a feeding needle in a total volume of 100 μl for 7 d prior to infection with their respective control groups. All the animals were observed for any clinical symptoms during the entire period of the experiment. Blood was collected via tail vein to evaluate potential hepatotoxic effect using GPT ELISA kit and cytokine production that are important in the outcome of *Brucella* infection using BD CBA mouse inflammation kit according to the manufacturers’ protocols. Mice were then intraperitoneally infected with *B. abortus* at 2 × 10^4^ CFU in 100 μl PBS. Oral treatment was continued until 14 d post-infection. Blood was collected at 7 and 14 d post-infection to evaluate cytokine production, and the mice were sacrificed at 15 d post-infection via cervical dislocation. Livers and spleens were aseptically collected, weighed and 0.05 g part was collected for CFU per g organ determination. A part of the organ was homogenized in 1 ml PBS, further diluted, plated on *Brucella* agar and then incubated for 3 d.

### Statistical Analysis

The results of data were expressed mean ± standard deviation (SD). At least triplicate samples from at least three independent experiments were used for in vitro experiments. Animal groups were consisted of at least six mice per group. GraphPad InStat software version 3 (GraphPad Software, Inc., USA) using Student’s *t*-test was used to compare results between two groups and the statistical difference was set at *p* < 0.05.

## Results

### Influence of LOX Inhibitors on the Viability of RAW264.7 Cells

**Fig. 1:**
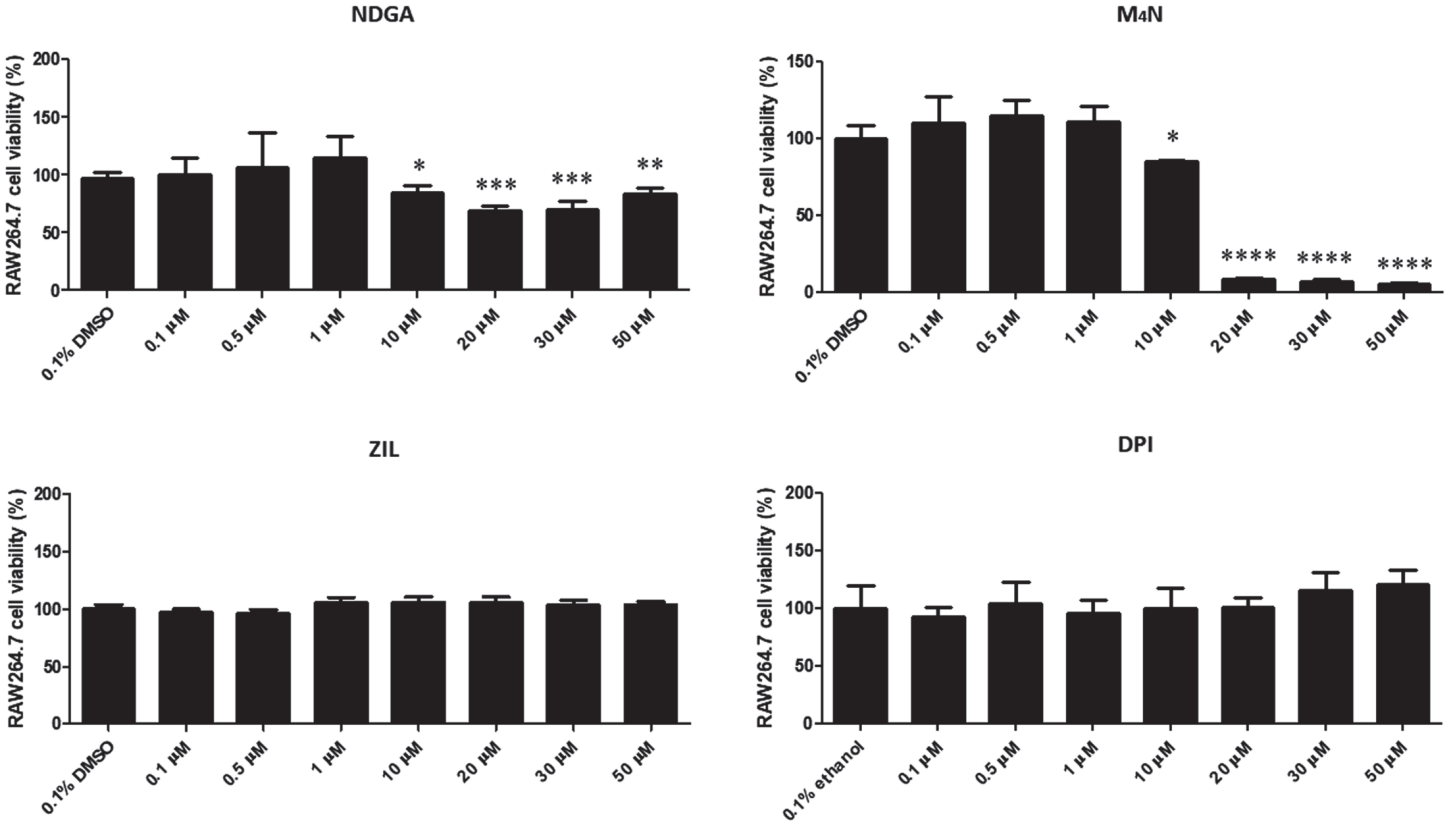
Effect of LOX inhibitors on the viability of RAW264.7 macrophages. Cells were incubated with different concentrations of NDGA, M_4_N, ZIL or DPI for 48 h. Data represent the mean ± SD. Statistically significant differences relative to the vehicle control are indicated by asterisks (*, *p* < 0.05; **, *p* < 0.01; ***, *p* < 0.001; ****, *p* < 0.0001).

RAW264.7 cells were incubated at different concentrations of LOX inhibitor NDGA (0, 0.1, 0.5, 1, 10, 20, 30, and 50 μM), its derivative M_4_N (0, 0.1, 0.5, 1, 10, 20, 30, and 50 μM) and 5-LOX inhibitor ZIL (0, 0.1, 0.5, 1, 10, 20, 30, 50 μM) for 48 h. Furthermore, overnight culture of cells were incubated at different concentrations of an unrelated inhibitor from COX pathway, TXA_2_ inhibitor DPI (0, 0.1, 0.5, 1, 10, 20, 30, 50, 60, 70, 80, 90, 100, 200, 300 μM). The results indicate that the highest non-cytotoxic concentration of NDGA and M_4_N was 1 μM (Fig. 1). On the other hand, 50 μM was the highest non-cytotoxic concentration for ZIL and DPI (Fig. 1). Hence these concentrations were used for the succeeding experiments including the in vivo tests.

### Influence of LOX Inhibitors on the Internalization of *B. abortus* into RAW264.7 Cells

**Fig. 2:**
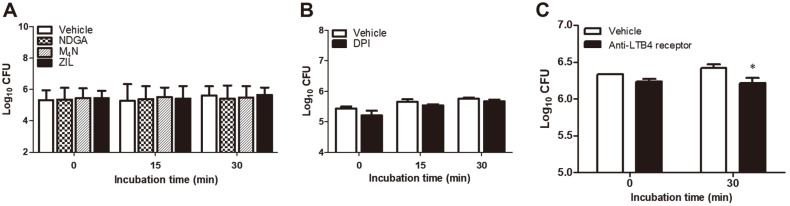
Effect of LOX inhibitors on the uptake of *B. abortus* to RAW264.7 macrophages. Cells were pre-incubated with NDGA (1 μM), M_4_N (1 μM), ZIL (50 μM) or DPI (50 μM) for 4 h. After washing, cells were infected with *Brucella* and incubated for 0, 15 and 30 min. The bacterial CFUs per ml was determined in LOX inhibitors (**A**), TXA_2_ inhibitor (**B**) and blocking LTB4 receptor (**C**). Data represent the mean ± SD. Statistically significant differences relative to the vehicle control are indicated by asterisk (*, *p* < 0.05).

None of the LOX inhibitors affected the uptake of *Brucella* into macrophages at all time points tested (Fig. 2A). DPI treatment did not also affect the uptake to the bacteria into the cells (Fig. 2B). This compound is an imidazole derivative that has been reported to block the formation of TXA_2_ from PG endoperoxides via COX pathway [19, 20]. The results suggest that these compounds that are known inhibitors in the LOX and COX pathways had no influence in the phagocytic pathway of *Brucella*. On the other hand, we also preliminary tested the effect of neutralizing LTB4 receptor by using a polyclonal antibody (1 μg/ml) targeting BLT2 in the uptake of the bacteria into RAW264.7 cells at 0 and 30 min post-infection and the results showed that lower number of bacteria entered the cells at 30 min post-infection (Fig. 2C). LTB4 is a product of the 5-LOX pathway of AA metabolism and is known as an important proinflammatory mediator, a potent chemotactic agent for neutrophils and other leukocytes, and promotes cell adhesion protein expression [21]. Although inhibitors of LOX and COX pathways did not affect the uptake of *Brucella* into macrophages, data suggests that LTB4 receptor, which is a GPCR, play a critical role in the internalization of *Brucella* into phagocytic cells.

### Influence of LOX Inhibitors on the Intracellular Growth of *B. abortus* Within RAW264.7 Cells

**Fig. 3:**
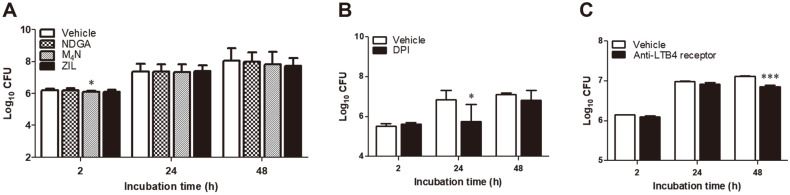
Effect of LOX inhibitors in the survival of *B. abortus* within RAW264.7 macrophages. Cells were infected with *Brucella* and after washing, incubated with NDGA (1 μM), M_4_N (1 μM), ZIL (50 μM) or DPI (50 μM) for a total of 2, 24 and 48 h. The bacterial CFUs per ml was determined in LOX inhibitors (**A**), TXA_2_ inhibitor (**B**) and blocking LTB4 receptor (**C**). Data represent the mean ± SD. Statistically significant differences relative to the vehicle control are indicated by asterisks (*, *p* < 0.05; ***, *p* < 0.001).

Among the LOX inhibitors, only M_4_N treatment reduced the intracellular survival of *Brucella* in macrophages (Fig. 3A). The reduction was observed at 2 h post-incubation while no significant differences were observed in the succeeding time points. DPI treatment showed lower intracellular growth of the bacteria at 24 h post-incubation (Fig. 3B). Neutralizing LTB4 receptor showed reduced number of bacteria that survived inside macrophages at 48 h post-incubation (Fig. 3C). This could suggest that inhibiting 5-LOX affects the intracellular signaling pathway of *Brucella* at early time point of infection. Nevertheless, in the context of COX pathway particularly inhibiting TXA_2_ could also be involved in the survival of *Brucella* inside its host cell. Blocking LTB4 receptor effectively inhibited the survival of *Brucella* within RAW264.7 cells at later time point of infection suggesting its role in the control of infection at a later stage.

### Influence of LOX Inhibitors in the Proliferation of *B. abortus* in the Organs of ICR Mice

**Fig. 4:**
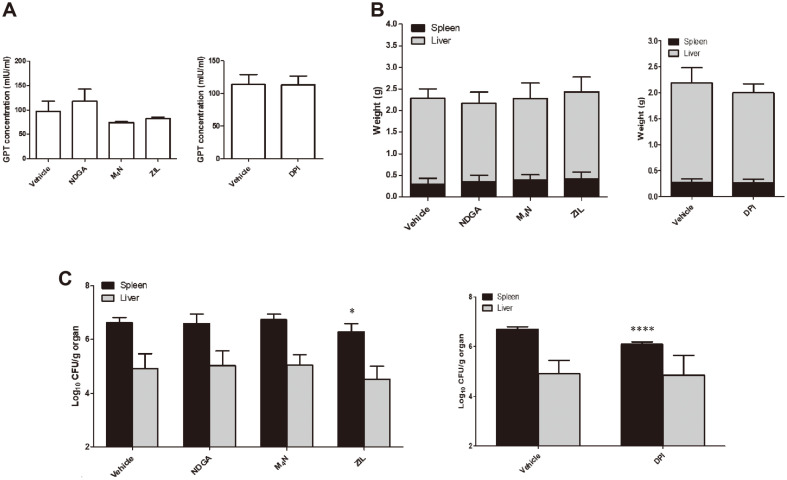
Effect of oral treatment of LOX inhibitors in proliferation of *Brucella* in the spleens and livers of ICR mice. Seven-week-old female ICR mice were acclimatized for one week prior to treatment. Mice were orally treated using a feeding needle with NDGA (1 μM), M_4_N (1 μM), ZIL (50 μM) or DPI (50 μM) for seven days with their respective control groups. Blood was collected via tail vein and serum was evaluated to evaluate potential hepatotoxic effect of the treatment (**A**). Mice were then intraperitoneally infected with *Brucella* (at 2 x 10^4^ CFU in 100 μl PBS). Oral treatment was continued until 14 d post-infection and the mice were sacrificed at 15 d post-infection. The weights of the spleens and livers were determined (**B**) and the log_10_ CFU/g organ was analyzed (**C**). Data represent the mean ± SD. Statistically significant differences relative to the vehicle control are indicated by asterisks (*, *p* < 0.05; ****, *p* < 0.0001).

Mice were monitored for any clinical symptoms and at 7 d post-treatment, blood was analyzed for GPT level and showed no differences among treatment groups as compared to their respective control groups indicating that the animals were in normal condition during the treatment period (Fig. 4A). The total average spleen and liver weights of all the treatment groups displayed no differences with their respective control groups (Fig. 4B) as well as the proliferation of *Brucella* into the organ liver (Fig. 4C). However, mice that received oral treatment of ZIL and DPI displayed significant reduction of the number of bacteria in the spleens at 15 d post-infection (Fig. 4C). Liver and spleen are the most affected organs in mice infected with *Brucella* but spleen has been reported to have higher number of bacteria per gram and the peak of infection was reached at two weeks post-infection [22]. Overall, the results suggest that 5-LOX and COX pathways could be involved in the proliferation of *Brucella* in mice.

### Effect of LOX Inhibitors Treatment in Mice in the Production of Cytokines

**Fig. 5:**
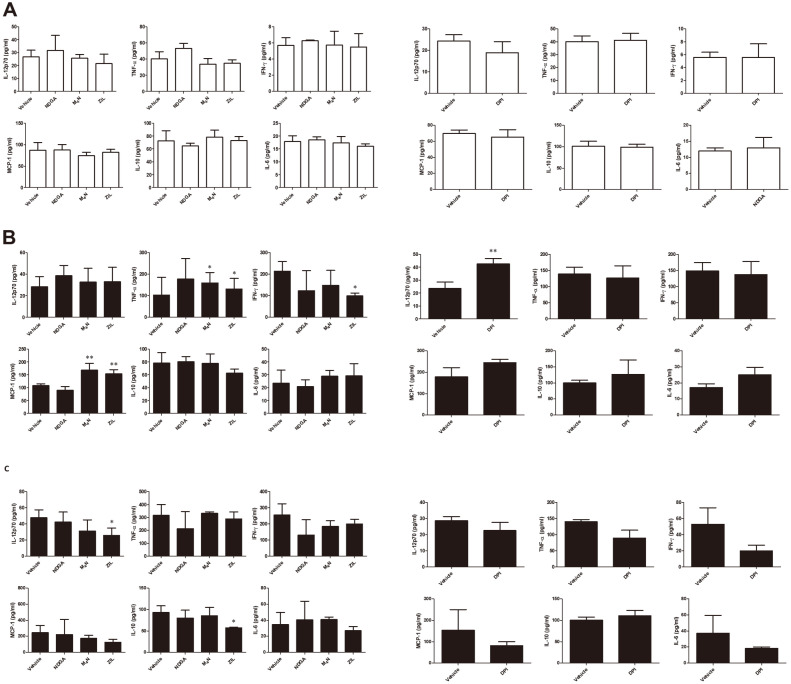
Effect of oral treatment of LOX inhibitors in the production of cytokines in ICR mice. Mice were orally treated with NDGA (1 μM), M_4_N (1 μM), ZIL (50 μM) or DPI (50 μM) for 14 d. The serum cytokine levels were determined in mice treated with LOX inhibitors or TXA_2_ inhibitor at 7 d (**A**), 7 d post-infection (**B**) and 14 d post-infection (**C**). Data represent the mean ± SD. Statistically significant differences relative to the vehicle control are indicated by asterisks (*, *p* < 0.05; **, *p* < 0.01).

Oral treatment of mice for one week with NDGA, M_4_N, ZIL or DPI did not affect the serum cytokine level of IL-12, TNF-α, IFN-γ, MCP-1, IL-10 and IL-6 as compared with their respective control group (Fig. 5A). At 7 d post-infection, M_4_N or ZIL-treated mice displayed increased serum level of TNF-α and MCP-1 while a lower serum level of IFN-γ was observed in ZIL-treated mice (Fig. 5B). Serum level of IL-12 was observed to be higher in mice treated with DPI at 7 d post-infection (Fig. 5B). At 14 d post-infection, serum levels of IL-12 and IL-10 were observed to be lower in ZIL-treated mice (Fig. 5C) while none of the remaining groups showed any differences in the serum cytokine production as compared to their respective control groups (Fig. 5C).

## Discussion

Brucella uses various strategies to modulate host immune response and has the capacity to survive as well as to replicate inside its host cells hence able to produce and establish a persistent and chronic infection [23]. AA metabolism-derived leukotrienes negatively regulates a protective Th1 immune response against bacterial infections via 5-LOX and *B. abortus* has been shown to activate 5-LOX responsible for the synthesis of these leukotrienes [24, 25]. Th1 immune response is important for host defense against intracellular pathogens such as *Brucella* although Th1 and Th2 immunity are not strictly synonymous with cell-mediated and humoral immunity since the former can stimulate moderate levels of antibody production while the latter can actively suppress phagocytosis [26]. Induction of AA production by *B. abortus*-infected dendritic cells was also reported [25]. On the other hand, we previously reported that AA had bactericidal effect against *B. abortus* [8]. Leukotrienes are formed via LOX pathway initiated by 5-LOX while PG, PC and TX are derived from AA via COX pathway [9]. Here we investigated the effects of LOX inhibitors such as 5-LOX inhibitors as well as an inhibitor in the COX downstream pathway. We also determined the effect of blocking a GPCR namely LTB4 receptor. Blocking 5-LOX nor TXA_2_ production did not alter the uptake of *Brucella* into macrophages suggesting that LOX and COX pathways might not be involved in the phagocytic pathway of *Brucella*, however further investigations are needed since limited inhibitors were used in the present study. Interestingly, blocking LTB4 receptor affected internalization of *Brucella* indicating other mechanisms involved particularly the positive role of GPCRs in the phagocytosis of the pathogen into host cells. LTB4 signaling via BLT receptors, particularly BLT1 which are mainly expressed by leukocytes such as neutrophils and eosinophils, mediates chemoattractive properties and proinflammatory effects and activates several kinase cascades leading to transcription of cytokine genes [27]. However, in the present study, neutralizing BLT2 significantly attenuated *Brucella* uptake at 30 min post-infection suggesting an important role of this receptor during the infection. BLT2 is typical GPCR with seven transmembrane helices, shares modest homology with BLT1 and has been reported to be expressed ubiquitously in human tissues and highly expressed in mouse epithelial cells including of the small intestine, colon and skin [27, 28]. Although LTB4 is a low-affinity receptor for BLT2, several eicosanoids are able to activate this receptor but not BLT1 indicating the role of inhibiting other specific high-affinity endogenous ligand for BLT2, such as 12(S)-hydroxy-5Z,8E,10E-heptadecatrienoic acid (12-HHT), that are involved in the progression of *Brucella* infection. Ishii and colleagues [29] reported that 12-HTT/BLT2 axis enhances epithelial barrier function and functions via Gαi protein-p38 MAPK pathway which might explain the inhibitory effect of blocking BLT2 receptor in the phagocytic pathway of *Brucella*. On the other hand, in response to different types of stimuli such as cytokines, PLA2 is activated to produce AA from membrane phospholipids and these free AA can be metabolized to other metabolic by-products by certain enzymes such as COX-2 [25]. Previously, we showed that COX-2 was significantly expressed in infected bone marrow-derived macrophages (BMDMs) from mice and RAW264.7 cells [8]. In the present study, inhibiting AA metabolism could be a protective approach against dissemination of *B. abortus* inside host cells.

Oral treatment of mice using the compounds that are reported to have an inhibitory effect either in LOX or COX pathway did not alter the production of different cytokines during no infection. NDGA and its derivative M_4_N did not affect the proliferation of *Brucella* in infected mice but a significant reduction of the number of bacteria was observed in mice that received an oral treatment of ZIL or DPI suggesting a different mechanism involved in the control of infection. However, there is a possibility that a higher dosage using either NDGA or M_4_N for the treatment of mice could give a protective effect against *Brucella*. ZIL is a specific 5-LOX inhibitor while DPI is a COX inhibitor particularly TXA_2_ production. In agreement to a study done by Fahel *et al*. [24], 5-LOX negatively regulates Th1 responses during *B. abortus* infection in mice and specific inhibition of this enzyme could be helpful in the control of these pathogens in animals. However, M_4_N-treated *Brucella*-infected mice at 7 d post-infection displayed increased serum level of TNF-α and MCP-1 suggesting its potential use as a vaccine adjuvant. These two cytokines were also observed to be higher in ZIL-treated mice suggesting the role of these cytokines in the control of *Brucella* dissemination in the mice. TNF-α plays a critical role in the activation of macrophages and is required for an effective clearance of *Brucella* infection while MCP-1 is reported to be important for antiviral immune responses and observed to play a role in the control of *B. abortus* infection [30-32]. Mice treated with DPI displayed increased serum level of IL-12 at 7 d post-infection suggesting its positive role in the proliferation of *Brucella* in the spleens of these mice. IL-12 is pivotal for Th1 responses development and mice depleted with this cytokine revealed to be more susceptible to *B. abortus* infection [33]. Interestingly, IFN-γ serum level was observed to be lower in mice that treated with ZIL suggesting its proinflammatory effect but in a study done by Kim *et al*. [34], transient production of this cytokine during the placental developmental period induces *B. abortus*-induced abortion in ICR mice indicating the promising benefit of ZIL in the prevention of abortion in animals although more investigations are necessary to prove this claim. However, IFN-γ/IL-10 ratio in mice treated with ZIL at 7 d post-infection was greater than 1 indicating a favorable Th1 immune response which is known to be important for the control of intracellular pathogens. Furthermore, lower serum levels of IL-12 and IL-10 were observed in ZIL-treated mice at 14 d post-infection. Interestingly, IFN-γ/IL-10 ratio still remained higher than 1 and more profound than observed at 7 d post-infection. IL-12 is a potent immunoregulatory cytokine and in a study done by Haraguchi *et al*. [35], the addition of recombinant IFN-γ enhanced IL-12 production. IL-12 although induces IFN-γ production by T and NK cells hence promoting a Th1 type immune response, in turn augments IL-12 production [36] which might explain the sudden reduction of IL-12 production where IFN-γ production was observed to be lower in mice treated with ZIL. Since IL-12 has also been linked with autoimmune inflammatory diseases associated with IFN-γ, the present study suggested the potential application of ZIL treatment for the control of autoimmune disorder. IL-10, on the other hand, reported to modulate the balance between the clearance of pathogen and immunopathology while the lack of this cytokine increases resistance to *Brucella* infection and its early induction in *B. abortus*-infected monocytes favors infection [37, 38]. Hence, decreased IL-10 serum level in ZIL-treated mice could be beneficial for the control of *Brucella* infection in these animals. It is noteworthy the potential immunomodulatory effect of ZIL and DPI during early infection at 7 d post-infection in the control of *Brucella* infection. ZIL treatment suggests potential ability to prevent abortion at early time point of infection while promoting Th1 immune response at the peak of infection. On the other hand, DPI treatment suggests a different role in the control of brucellosis aside from immunomodulation which encourages further investigations. Taken together, inhibition of 5-LOX and TXA_2_ or a combination therapy could be a potential alternative therapy against *B. abortus* infection as well as other intracellular pathogens.
